# Effect of Optimal Daily Fertigation on Migration of Water and Salt in Soil, Root Growth and Fruit Yield of Cucumber (*Cucumis sativus* L.) in Solar-Greenhouse

**DOI:** 10.1371/journal.pone.0086975

**Published:** 2014-01-27

**Authors:** Xinshu Liang, Yinan Gao, Xiaoying Zhang, Yongqiang Tian, Zhenxian Zhang, Lihong Gao

**Affiliations:** Beijing Key Laboratory of Growth and Developmental Regulation for Protected Vegetable Crops, Department of Vegetable Science, China Agricultural University, Beijing, P.R. China; Institute of Genetics and Developmental Biology, Chinese Academy of Sciences, China

## Abstract

Inappropriate and excessive irrigation and fertilization have led to the predominant decline of crop yields, and water and fertilizer use efficiency in intensive vegetable production systems in China. For many vegetables, fertigation can be applied daily according to the actual water and nutrient requirement of crops. A greenhouse study was therefore conducted to investigate the effect of daily fertigation on migration of water and salt in soil, and root growth and fruit yield of cucumber. The treatments included conventional interval fertigation, optimal interval fertigation and optimal daily fertigation. Generally, although soil under the treatment optimal interval fertigation received much lower fertilizers than soil under conventional interval fertigation, the treatment optimal interval fertigation did not statistically decrease the economic yield and fruit nutrition quality of cucumber when compare to conventional interval fertigation. In addition, the treatment optimal interval fertigation effectively avoided inorganic nitrogen accumulation in soil and significantly (*P*<0.05) increased the partial factor productivity of applied nitrogen by 88% and 209% in the early-spring and autumn-winter seasons, respectively, when compared to conventional interval fertigation. Although soils under the treatments optimal interval fertigation and optimal daily fertigation received the same amount of fertilizers, the treatment optimal daily fertigation maintained the relatively stable water, electrical conductivity and mineral nitrogen levels in surface soils, promoted fine root (<1.5 mm diameter) growth of cucumber, and eventually increased cucumber economic yield by 6.2% and 8.3% and partial factor productivity of applied nitrogen by 55% and 75% in the early-spring and autumn-winter seasons, respectively, when compared to the treatment optimal interval fertigation. These results suggested that optimal daily fertigation is a beneficial practice for improving crop yield and the water and fertilizers use efficiency in solar greenhouse.

## Introduction

Protected vegetable production systems have been rapidly developed in recent decades in China. Solar-greenhouse, an unheated plastic greenhouse using solar light energy to make sure that vegetables grow normally, plays more and more important roles in China's vegetable production and supplication during the winter [1]. However, excessive irrigation and fertilization are commonly practiced in the solar-greenhouse production systems. For instance, some investigations have revealed that irrigation water rate is 1000 mm each year and fertilizer N apparent recovery efficiency can be less than 10% using conventional management practices [2], [3]. Consequently, redundant water and fertilizers affect environmental protection by nutrient accumulation and soil salinization [4]–[6].

It has become the focus of agricultural field that the efficient water and fertilizer management methods are applied in vegetable production in greenhouse. For example, Cabello et al. [7] demonstrated that under moderate deficit irrigation (90% evapotranspiration) condition, reducing the inputs of nitrogen fertilizer did not reduce yield of melon, but increased water and nitrogen fertilizer use efficiencies. Similarly, Mahajan and Singh [8] found that fertigation through reducing rates of water and nitrogen fertilizer promoted fruit yield and quality, and water and fertilizer use efficiencies in greenhouse tomato cultivation system. In addition, both subsurface drip irrigation and alternate furrow irrigation can significantly improve the root growth, and thus enhance the yield and water and fertilizer use efficiencies [9]–[12].

However, conventional interval fertigation is still common in intensive vegetable production systems in China. In general, the period between successive irrigation and fertilization events is 7 to 10 days, or even longer. Consequently, the nutrient concentration in the root-zone soil may be in excess of plant requirement for growth on the first day after fertigation, and then decreases gradually to reach deficit levels before the day preceding the next fertigation event, and eventually inhibit crop growth and development [13]. Therefore, there is a need for ensuring water and nutrient in root-zone soil stable to reduce adverse effects caused by their fluctuation on crop growth under intensive vegetable management practices.

Drip fertigation, a technique used to manage soil water and nutrition supplies according to the actual water and nutrient requirement of the plants, can be applied to vegetable production to maintain stable water and nutrition contents in the root zone of crops. It applies frequent and small amounts of soluble fertilizers along with water by exerting the soil buffer characteristic and by reducing the time interval between successive irrigations [14], [15]. This technique is still rarely implemented in soil cultivation systems in developing countries, despite of the fact that it has been applied for many years in developed countries.

The efficiency of a fertigation treatment can be evaluated with a combination of the availability and distribution of soil water and nutrients, the growth and development of crop root, and the formation of crop yield. These parameters are relatively simple, rapid, easy to obtain and cost effective. Among these parameters, particular attention should be paid to plant root since the morphology and spatial configuration of root can significantly affect soil water and nutrient transformation, mobilization and use efficiency by plant and crop yield [16]–[18]. However, due to the complexity of root growth environment and the limitations of root research methods, little information is available regarding the vegetable root growth under daily fertigation based on crop requirement under greenhouse soil cultivation conditions.

Cucumber is one of the major greenhouse vegetables in China. However, inappropriate irrigation and fertilizing practices have caused soil nutrient imbalance that negatively affects cucumber growth and the reduction of soil water and fertilizer use efficiencies. It is important for farmers to manage soil water and nutrition supplies according to the actual water and nutrient requirement of the plants during greenhouse cucumber production seasons. Thus, the objectives of this study were to investigate how the migration of water and salt in soil, and root growth and fruit yield of cucumber are affected by the daily fertigation based upon the actual water and nutrient requirement of the plants.

## Materials and Methods

### Ethics statement

No specific permissions were required for the described field study.The experiment was carried out in 2011 in a typical solar greenhouse of Fangshan District Agricultural Science Research Institute, Beijing, China (39.7°N; 116.1°E), which is an experimental station of China Agricultural University. The field study did not involve endangered or protected species.

### Site description and experimental design

**Figure 1:**
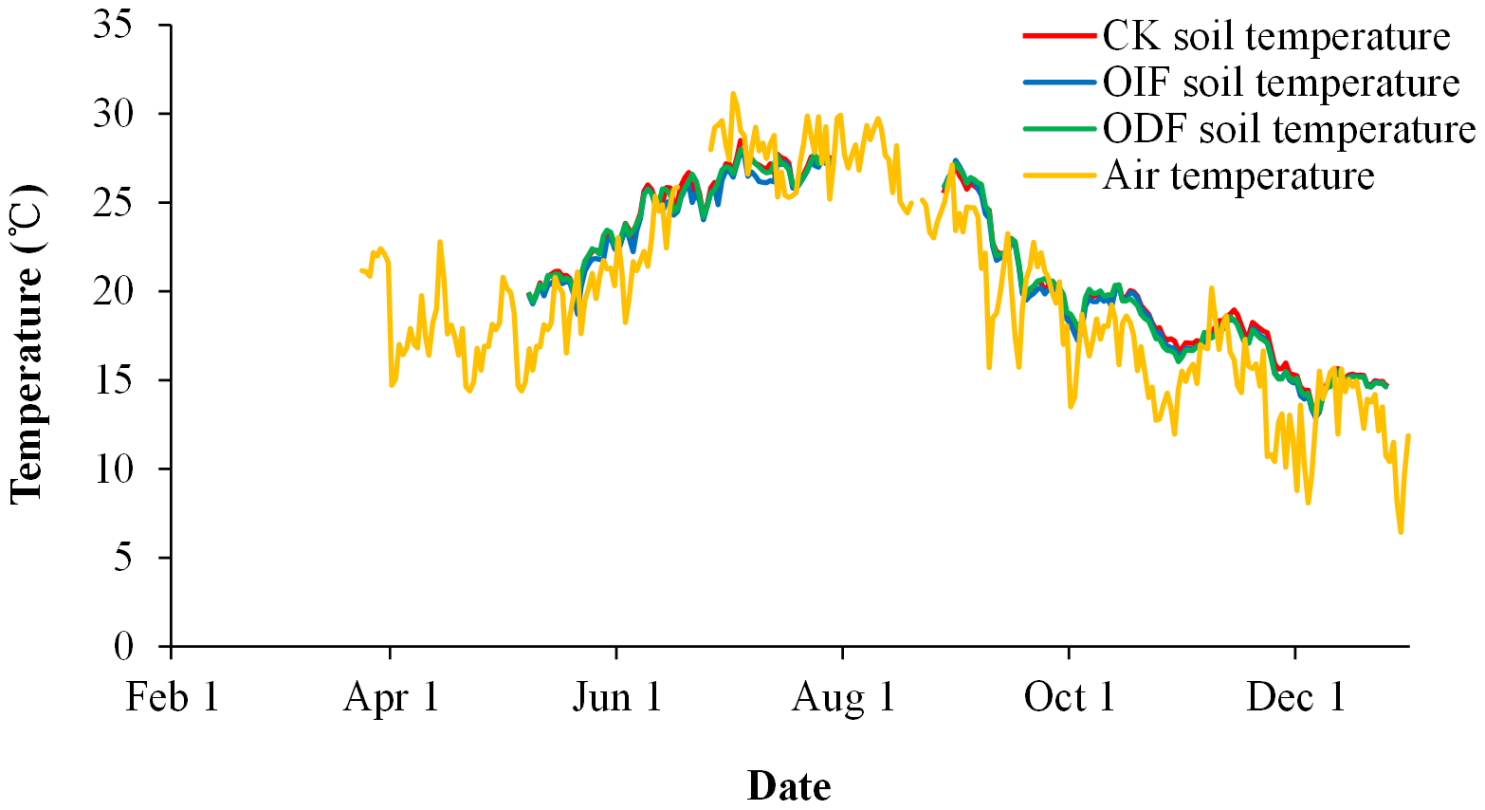
Daily average soil temperatures at 10(CK), optimal interval fertigation (OIF) and optimal daily fertigation (ODF) and daily average air temperature at 150 cm height in cucumber greenhouse cropping system in 2011 at Fangshan, Beijing suburbs. These data were determined by RTH-1010 TPE rensin-shield sensor and RT-12 Thermo Recorder made in Japan. Time interval for data recording was set to 10 minutes.

The greenhouse was covered with polyethylene film (ground area 56 m×7 m) without supplementary lighting and heating. Daily average soil temperatures and air temperature in solar-greenhouse were shown in Fig. 1. Both the two temperatures in April gradually increased until each reached the highest value in July and August, and then gradually decreased. Furthermore, Daily average soil temperatures at 10 cm depth from conventional interval fertigation (CK), optimal interval fertigation (OIF) and optimal daily fertigation (ODF) were not significantly different. Field experiments were conducted on a sandy loam soil (36% sand, 48% silt and 16% clay). The topsoil (0–30 cm layer) had a pH (1∶2.5 soil/water) value of 7.37, an electrical conductivity (EC) (1∶5 soil/water) value of 1.17 dS m^−1^, a field capacity of 20.8% and a bulk density of 1.36 g cm^−3^, and contained 15.8 g kg^−1^ organic matter, 0.96 g kg^−1^ total nitrogen (N), 195 mg kg^−1^ inorganic N, 193 mg kg^−1^ available phosphorus (P) and 275 mg kg^−1^ available potassium (K).

The experimental period comprised two growth cycles including the early-spring (ES) (February 1-July 29) and autumn-winter (AW) (August 1-December 25) seasons. The varity of cucumber was Zhongnong No. 26. This varity was a high-quality and largely fruit-bearing cucumber hybrid. The average fruit length and width of this varity were 30 and 3.3 cm, respectively. Due to its high resistance to several diseases (including powdery mildew, downy mildew and gray mold) and tolerance to both low temperature and weak light, this varity was very suitable for cultivation in solar-greenhouse. Cucumber seedlings with two leaves were transplanted by hand, before which, soils were incorporated with organic fertilizer (basal fertilizer) at designed rates (Table 1), and were ploughed and harrowed to a depth of 30 cm. Plant pruning was performed as follows: all lateral branches were removed by hand, however, the axial shoot of cucumber was remained and it climbed upward along a vertical rope. Planting density of cucumber was 5 plants m^−2^.

**Table 1:** Amounts of fertilizers used in the treatments CK (conventional interval fertigation), OIF (optimal interval fertigation) and ODF (optimal daily fertigation) in the ES (early-spring) and AW (autumn-winter) seasons.

Cropping season	Treatment	Organic manure^a^	Chemical fertilizer (kg ha^−1^)			Irrigation water
		(t ha^−1^)	N	P_2_O_5_	K_2_O	(mm)
ES	CK	45.0	819.0	690.4	1006.2	349.2
	OIF	22.5	427.5	361.3	524.1	349.2
	ODF	22.5	427.5	361.3	524.1	349.2
AW	CK	60.0	465.0	312.0	593.4	147.2
	OIF	30.0	151.5	123.1	183.1	147.2
	ODF	30.0	151.5	123.1	183.1	147.2

a For the ES season, total N, total P and total K contents of the organic manure were 2.09% (N), 2.06% (P_2_O_5_) and 1.34% (K_2_O), respectively. For the AW season, total N, total P and total K contents of the organic manure were 1.30% (N), 0.5% (P_2_O_5_) and 1.06% (K_2_O), respectively.

**Figure 2:**
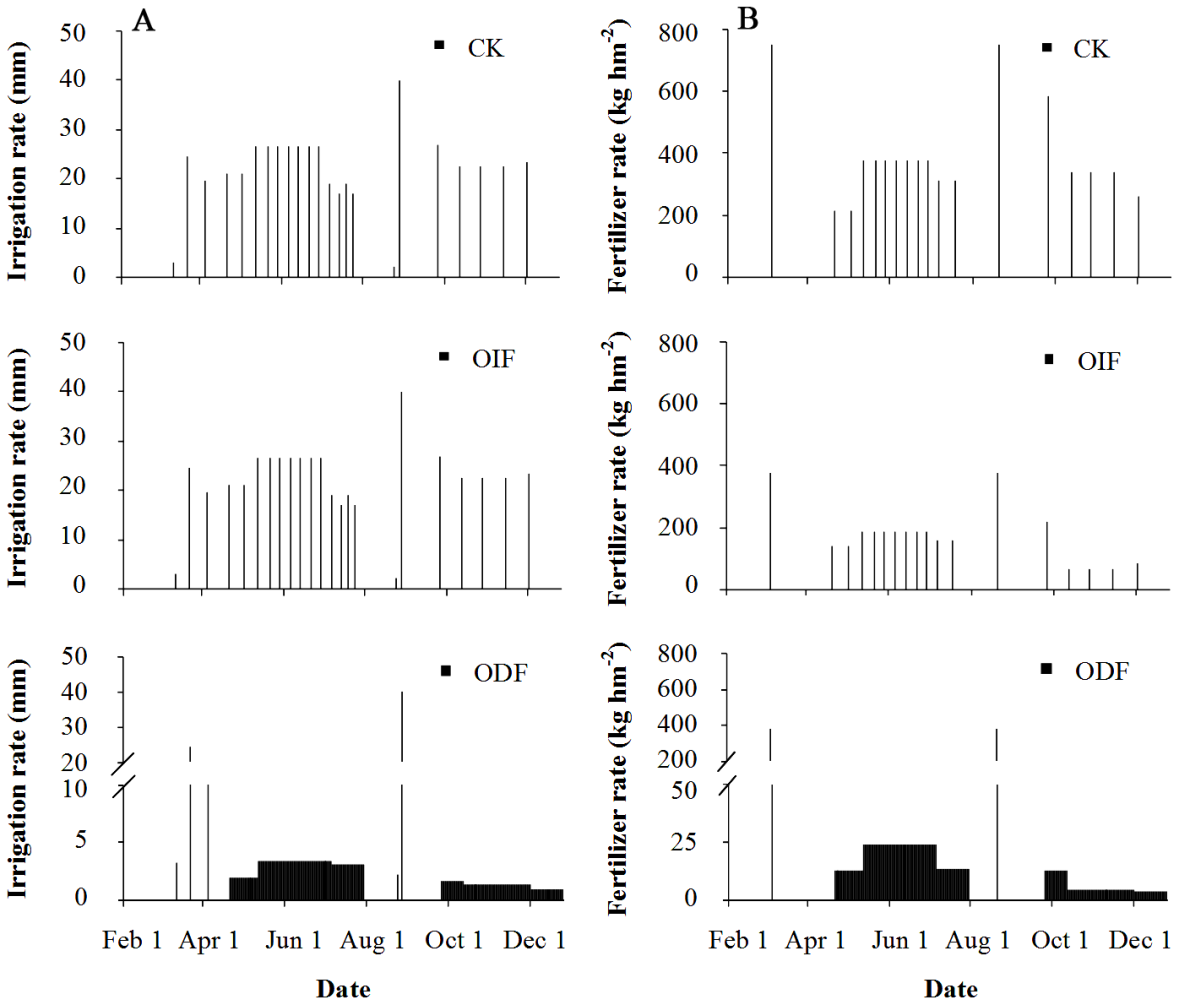
Irrigation scheduling (A) and fertilization scheduling (B) under three different fertigation ways in a solar greenhouse cucumber cultivation system. All fertilizer varity were compound fertilizer. The two N:P_2_O_5_:K_2_O formulations of basal fertilizer were respectively (18∶46∶0) and (15∶15∶15) during ES and AW seasons. The three N:P_2_O_5_:K_2_O formulations of topdressing fertilizer were respectively (20∶20∶20), (19∶8∶27) and (18∶6∶34) for early fruit stage, middle fruit stage, late fruit stage during ES and AW seasons.

The experiment consisted of three treatments:

Conventional interval fertigation (CK): Conventional organic manure (basal fertilizer) and chemical fertilizer were applied at rates based on average fertilization level used by greenhouse cucumber growers in the suburb of Beijing (Table 1). A detailed description of chemical fertilizer topdressing/irrigation rates under the CK was given in Fig. 2.
Optimal interval fertigation (OIF): The organic manure (basal fertilizer) application rate was half the rate conventionally applied per hectare (Table 1). Based on the N requirement for cucumber growth [19]–[21] and N fertilizer recommendation, the total N rates applied by topdressing were 427.5 and 151.5 kg N ha^−1^ in the ES and AW seasons (Table 1), respectively. These total mineral N (N_min_) application rates were calculated using the method of soil N balance, where expected yield of solar greenhouse cucumber were 120 and 60 t ha^−1^ in the ES and AW seasons, respectively. The equation [22] was as follows:

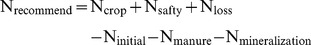
(1)Where N_recommend_ is Recommended fertilizer N; N_crop_ is crop N uptake; N_safty_ is soil N_min_ safety margin; N_loss_ is N loss; N_initial_ is soil N_min_ in the root zone before transplanting; N_manure_ is N_min_ from the mineralization of organic fertilizer; N_mineralization_ is N_min_ from the mineralization of organic nitrogen in soil.
  Chemical fertilizer topdressing/irrigation frequencies and irrigation rates were the same as the CK, except fertilizer topdressing rates. A detailed description of chemical fertilizer topdressing/irrigation rates under the treatment OIF was given in Fig. 2.
Optimal daily fertigation (ODF): The total amounts of organic manure (basal fertilizer) and chemical fertilizer were the same as treatment OIF (Table 1), however, chemical fertilizer was applied automatically daily according to the actual water and nutrient requirement of the plants. A detailed description of chemical fertilizer topdressing/irrigation rates under the treatment ODF was given in Fig. 2.

The total irrigation amount was the same for three treatments in whole growth period of cucumber. Fertigation was used under transparent thin film for all treatment. Irrigations were applied 16 and 7 times in the ES and AW seasons, respectively. The irrigation rate each time for CK and OIF teatments was 22.5–30 mm according to requirement rule of water for cucumber [23]–[25].

The chemical fertilizer used in this study was a special water-soluble fertilizer for cucumber called Shengdanshu (a local fertilizer). This fertilizer had three N:P_2_O_5_:K_2_O formulations, i.e., 20∶20∶20, 19∶8∶27 and 18∶6∶34, which were used at the early harvest stage (April 21 – May 12 during ES season, September 25 – October 11 during AW season), the middle harvest stage (May 13 – July 5 during ES season, October 12 – November 30 during AW season) and the late harvest stage (July 6 – July 29 during ES season, December 1 – December 25 during AW season), respectively. Fertilizers were applied 12 and 6 times in the ES and AW seasons, respectively. Except different fertigation methods related to different treatments, the same local management practices were applied in all treatments. The experiment was a randomized block design with four replications and the size of each replicate plot was 3.9 m×4.8 m. Each replicate plot had three cultivation furrows and was separated from the adjacent plots by plastic films buried at a depth of 50 cm.

### Soil water content, EC and mineral N content

To evaluate the migration of soil water and salt under different fertigation treatments, soil samples from five cores per subplot were collected five times within a single fertigation cycle during the middle fruit harvest period when daily fruit production was very high in each cropping season. For the ES season, soils were sampled on May 27, May 28, May 31, June 2 and June 4. For the AW season, the corresponding sampling times were October 26, October 27, November 1, November 6 and November 12. Soil samples were taken at 0–15, 15–30 and 30–45 cm depth. Soil samples of each plot at each depth were mixed thoroughly and passed through a 2-mm sieve. Sub-samples of 20 g fresh soil were dried for 12 h at 105°C, and then soil water content was determined at the ratio of water and dry soil weight. Sub-samples of about 300 g fresh soil used to measure EC were air-dried passed through a 1-mm sieve. Soil EC was analyzed from a 1∶5 (*w*/*v*) soil (air-dried) to water ratio using an EC meter and combination glass electrodes (FE30, METTLER TOLEDO, Shanghai, China). Sub-samples of 12 g fresh soil used to estimate mineral N were submerged into 100 ml 0.01 M CaCl_2_ solution and shaken for 1 hour to extract inorganic N. The extracts were filtered and analyzed using an continuous flowing analyzer (TRAACS2000, USA) to determine NO_3_^−^-N and NH_4_^+^-N contents [26].

### Cucumber root morphologic characters

Once the final harvest was completed, one typical cucumber root in each plot was collected with an Eijelkamp root auger (length  = 0.15 m, diameter  = 0.08 m) from the 0–15, 15–30 and 30–45 cm soil layers. For each layer, 9 holes were drilled around the cucumber main root in a shape of 3×3 cross square. The roots in the soil were carefully selected and washed to acquire the roots from different soil layers. Root morphology was analyzed by using fresh roots and a root scanner system (EPSON EXPRESSION 4990, Japan). Data were then analyzed with the WinRHIZO root analysis software (LC4800-II LA2400; Saint foy, Canada) to determine the root characteristics, including root length, root surface, root volume and average diameter. The root characteristics could be divided into four classification according to the root diameters aggregated into classes of 0.0–0.5, 0.5–1.0, 1.0–1.5 and ≥1.5 mm. The scanned roots were oven-dried at 65°C until weight constancy and weighed.

### Cucumber fruit yield, irrigation water use efficiency and partial factor productivity of applied nitrogen

Economic yield was measured for whole cucumber growth cycles in each plot and translated into economic yield weight per hectare. The ratio of yield to water supply was referred to as irrigation water use efficiency (*IWUE*, kg mm^−1^):
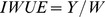
(2)where *Y* and *W* represent the economic yield (kg ha^−1^) and the amount of water (mm) applied to the cucumber during the growing cycle [7], respectively. The ratio of yield to N supply is referred to as partial factor productivity of applied N (*PFP_N_*, kg kg^−1^):
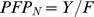
(3)where *F* is the amount of fertilizer N (kg) applied to the cucumber during the growing cycle [27].

### Vitamin C, soluble sugar and nitrate contents in cucumber fruit

To estimate cucumber fruit quality under different fertigation treatments, three quality parameters which were concerned by local residents were made. Fresh fruit samples of cucumber in each plot were collected when daily fruit production was very high, and their appearance should be similar and marketable. Fresh fruits were washed, chopped and mixed in the lab to assess different quality indexes. Contents of Vitamin C and soluble sugar were determined by the methods of 2, 6-dichloro-indophenol titration [28] and anthrone ethyl acetate colorimetic [29], respectively. Content of nitrate was measured by the method of sulfuric acid-acid [30].

### Statistical analysis

SPSS 17.0 was used to analyse data. Treatment means were separated using the least significant difference (LSD) test at *P*<0. 05. Principal component analysis (PCA) was done to comprehensively determine the whole effect of different fertigation methods on several root morphologic characters (Root dry weight, length, surface area, average diameter and volume).

## Results

### Soil water content, EC and mineral N content

**Figure 3:**
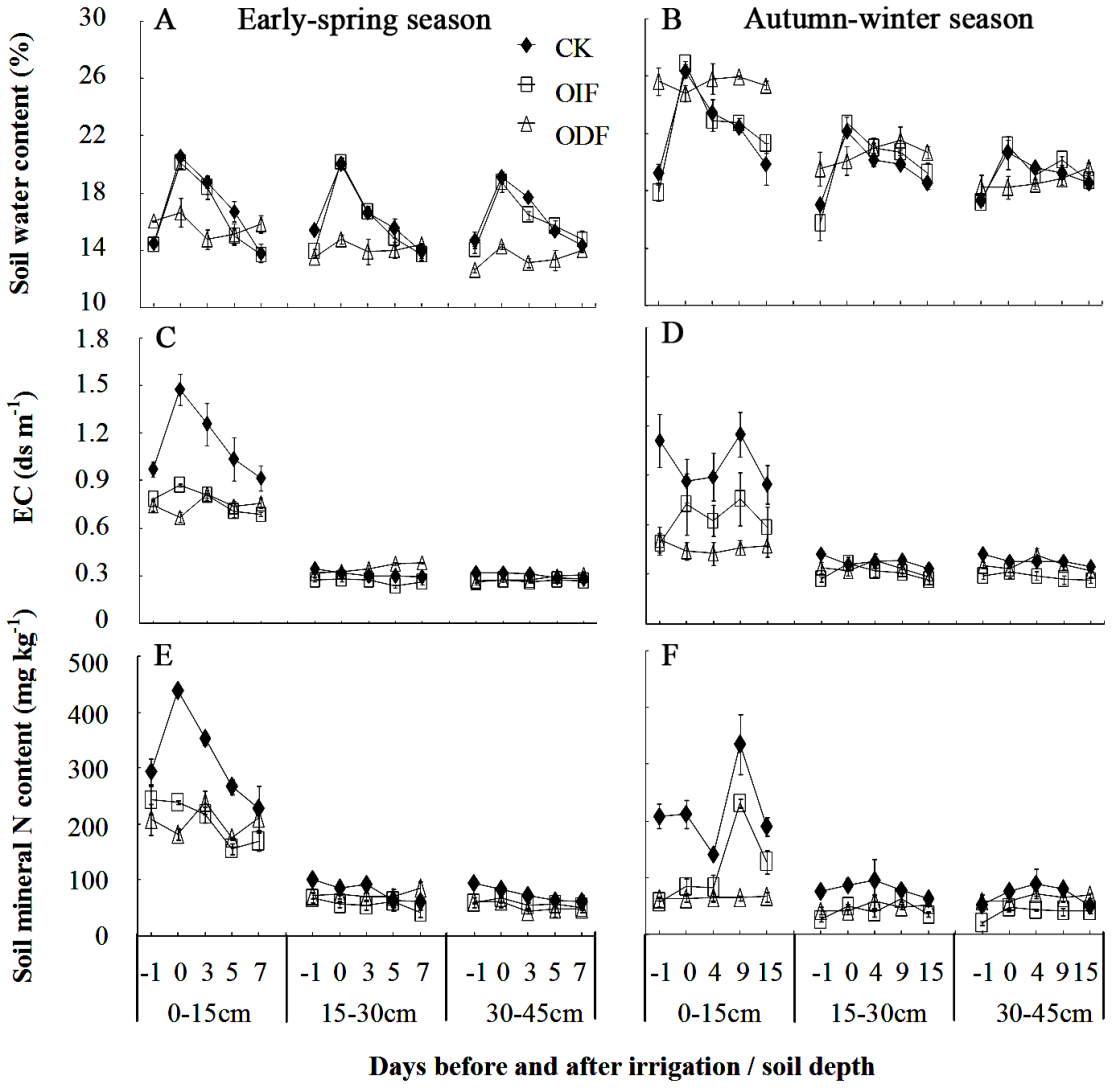
Effects of conventional interval fertigation (CK), optimal interval fertigation (OIF) and optimal daily fertigation (ODF) on changes of soil water content (A and B), EC value (C and D) and mineral N content (E and F) in the early-spring (ES) and autumn-winter (AW) seasons. The numbers on the abscissa represent the days before (negative value), during (zero) and after (positive value) irrigation. Bars represent standard errors.

Soil water content under the treatments OIF and CK increased rapidly and then decreased gradually within an irrigation cycle (Fig. 3). However, it maintained a relatively stable level under the treatment ODF. No significant difference was found in soil water content between the treatments OIF and CK in all tested soil layers in both ES and AW seasons. The treatment ODF decreased the amplitude (i.e. the difference between maximum and minimum values) of soil water content in all tested soil layers during ES season and that in the 0–15 cm soil layer during AW season in an irrigation cycle, when compared to the treatments OIF and CK (Table 2). In addition, the relative soil water content under the treatment ODF was higher in the 0–15 cm soil layer than in the 15–30 and 30–45 cm soil layers, suggesting that the soil water was concentrated by the treatment ODF in the 0–15 cm soil layer.

**Table 2:** Effects of conventional interval fertigation (CK), optimal interval fertigation (OIF) and optimal daily fertigation (ODF) on the amplitudes (the difference between maximum and minimum values) of soil water content, EC value and mineral N content in the early-spring (ES) and autumn-winter (AW) seasons.

Cropping season	Treatment	Soil water (%)			Soil EC (mS cm^−1^)			Soil mineral N (mg kg^−1^)		
		0–15 cm^a^	15–30 cm	30–45 cm	0–15 cm	15–30 cm	30–45 cm	0–15 cm	15–30 cm	30–45 cm
ES	CK	7.0 a^b^	6.1 a	5.0 a	0.717 a	0.066 a	0.071 a	230.7 a	59 a	43.9 a
	OIF	6.5 a	6.6 a	5.0 a	0.204 b	0.062 a	0.033 a	114.5 b	39.9 b	35.6 a
	ODF	3.4 b	2.2 b	2.1 b	0.154 b	0.087 a	0.077 a	91.6 b	32.6 b	41.8 a
AW	CK	8.4 a	5.2 a	3.8 a	0.682 a	0.108 a	0.094 a	205.2 a	77.5 a	76.1 a
	OIF	9.0 a	7.0 a	4.0 a	0.495 a	0.130 a	0.076 a	177.6 a	38.7 a	36.4 b
	ODF	3.5 b	4.0 a	2.2 a	0.223 b	0.112 a	0.097 a	23.9 b	29.6 a	25.2 b

a Soil sampling layers

b The same letter in the same data column denotes no significant difference (P≤0.05) by LSD.

The EC values in the 0–15 cm soil layer were higher and more variable than those in the 15–30 and 30–45 cm soil layers, suggesting the salts were concentrated in the 0–15 cm soil layer (Fig. 3). The CK showed the highest and most variable EC values in the 0–15 cm soil layer, resulting in the highest amplitude of soil EC value (Table 2). Generally, in the ES season, the treatments OIF and ODF did not show significant differences in EC values in the 0–15 cm soil layer. However, in the AW season, EC values under the treatment OIF were significantly higher and more variable than those under the treatment ODF in most sampling times. The soil EC values were not significantly different (*P*>0.05) between any two treatments in the 15–30 and 30–45 soil layers in both ES and AW seasons.

Similar to soil EC values, generally, soil mineral N (N_min_) contents in the 0–15 cm soil layer were higher and more variable than those in the 15–30 and 30–45 cm soil layers (Fig. 3). In addition, the CK had the higher and more variable soil N_min_ contents in the 0–15 cm soil layer. However, the amplitude of soil N_min_ content was significantly higher under the CK than under the treatments OIF and ODF in the 15–30 and 30–45 soil layers in ES and AW seasons, respectively (Table 2).

### Principal component analysis of root growth properties

Root dry weight, length, surface area, average diameter and volume were used to evaluate the plant root growth. Since there were statistical links among each other of these root properties (all P<0.05), we used the principal component analysis (PCA) to transform these correlated root variables (Table 3) into two principal components (i.e. PC_1_ and PC_2_) to obtain two princioal expressions as follow:
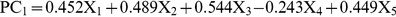
(4)

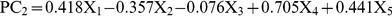
(5)where X_1_, X_2_, X_3_, X_4_ and X_5_ represent the root dry weight, length, surface area, average diameter and volume, respectively. The eigenvalue contribution rates of PC_1_ and PC_2_ were 65.95% and 31.60%, respectively.

**Table 3:** Effects of conventional interval fertigation (CK), optimal interval fertigation (OIF) and optimal daily fertigation (ODF) on the root weight, length, surface area, average diameter and volume in the early-spring (ES) and autumn-winter (AW) seasons.

Cropping season	Treatment	X_1_	X_2_	X_3_	X_4_	X_5_
		Root weight (g)	Root length (cm)	Root surface area (cm^2^)	Root average diameter (mm)	Root volume (cm^3^)
ES	CK	1.68 a^a^	4913.69 b	653.73 a	0.416 a	8.58 a
	OIF	1.86 a	5641.86 ab	732.78 a	0.407 a	9.31 a
	ODF	1.67 a	7368.76 a	781.54 a	0.370 b	8.47 a
AW	CK	1.51 a	6104.37 a	715.41 a	0.388 a	8.35 a
	OIF	1.09 b	3870.42 b	481.92 b	0.399 a	5.92 b
	ODF	1.31 a	5855.82 ab	659.25 a	0.384 a	7.08 b

a The same letter in the same data column denotes no significant difference (P≤0.05) by LSD.

Since the greater values of root dry weight, length, surface area and volume and the thinner root diameter, the root system is more powerful for absorption of water and nutrient, the comprehensive principal component (CPC) was received as follow:
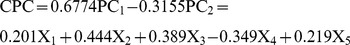
(6)


The treatment CK showed the lowest PC_1_ and CPC values in the ES season, but the highest PC_2_ values in the ES and AW seasons and PC_1_ and CPC values in the AW season (Table 4). However, the treatment OIF showed the lowest PC_1_ and CPC values in the AW season. The treatment ODF showed the lowest PC_2_ value in the ES and AW seasons, but the highest PC_1_ and CPC values in the ES season.

**Table 4:** Effects of conventional interval fertigation (CK), optimal interval fertigation (OIF) and optimal daily fertigation (ODF) on the first (PC1) and second (PC2) principal components and comprehensive principal component (CPC) values of root characteristic parameters in the early-spring (ES) and autumn-winter (AW) seasons.

Principal component	ES season			AW season		
	CK	OIF	ODF	CK	OIF	ODF
PC_1_	−0.09	1.05	1.61	0.50	−2.62	−0.46
PC_2_	1.28	1.26	−0.81	−0.22	−0.58	−0.93
CPC	−0.46	0.30	1.32	0.40	−0.93	−0.01

### Root length and distribution in different soil layers

**Figure 4:**
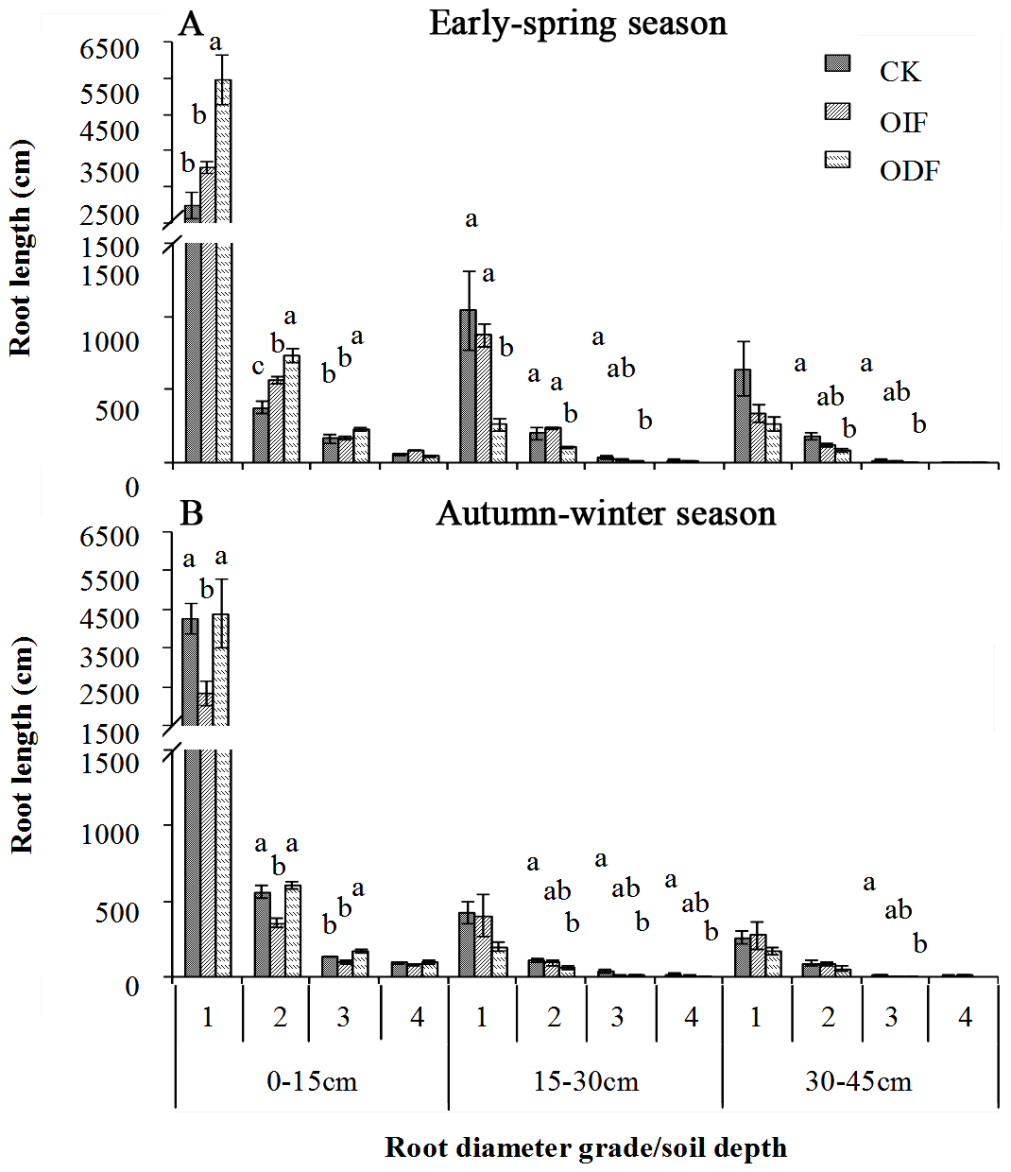
Effects of conventional interval fertigation (CK), optimal interval fertigation (OIF) and optimal daily fertigation (ODF) on the length and distribution of root with different diameter grades in different soil layers in the early-spring (A) and autumn-winter (B) seasons. The numbers 1, 2, 3 and 4 on the abscissa represent four root diameter scales, 0.0–0.5, 0.5–1.0, 1.0–1.5 and ≥1. 5 mm, respectively. Bars represent standard errors. The same letter in the same data column denotes no significant difference (P≤0.05) by LSD.

In general, root length of four diameter classes (i.e. 0.0–0.5, 0.5–1.0, 1.0–1.5 and >1.5 mm) decreased with rooting depth under all fertigation treatments (Fig. 4). However, while the fine roots (<0.5 mm diameter) mainly concentrated at 0–15 cm depth, the thicker roots (>0.5 mm diameter) were more evenly distributed with soil depth. Root length of 0.0–0.5 mm diameter was statistically affected by fertigation treatment within the uppermost 15 cm of the soil depth, however, root length of 0.5–1.5 mm diameter was statistically affected by fertigation treatment within the uppermost 30 cm of the soil depth (*P*<0.05; Fig. 4A,B). Root length of >1.5 mm diameter was only statistically affected by fertigation treatment in the 15–30 cm soil layer in the AW season (*P*<0.05; Fig. 4B). The treatment OIF significantly increased the root length of 0.5–1.0 mm diameter in the 0–15 cm soil layer in the ES season, however, the treatment ODF significantly increased the root length of 0.0–1.5 mm diameter in the 0–15 cm soil layer in the ES season and the root length of 1.0–1.5 mm diameter in the 0–15 cm soil layer in the AW season, when compared to the CK. Generally, the root lengths were not significantly different (*P*>0.05) between the treatment OIF and CK in the 15–30 and 30–45 soil layers in both ES and AW seasons. However, the treatment ODF significantly decreased the root length in the 15–30 and 30–45 soil layers in both ES and AW seasons, when compared to the CK.

### Cucumber economic yield, IWUE and PFP_N_

In both ES and AW seasons, cucumber economic yield, IWUE, PFP_N_ from the treatment ODF were significantly (P<0.05) higher than those from the CK (Table 5). No significant (P>0.05) difference was found in both cucumber economic yield and IWUE between the treatments OIF and CK, however, PFP_N_ from the treatment OIF was significantly (P<0.05) higher than that from the CK in both ES and AW seasons. The treatment ODF increased cucumber economic yield, IWUE and PFP_N_ by 6.2%, 6.1% and 103% in the ES season, and 8.3%, 8.4% and 232% in the AW season when compared to the CK, respectively. The treatment OIF increased PFP_N_ by 88% and 209% in the ES and AW seasons when compared to the CK, respectively.

**Table 5:** Effects of conventional interval fertigation (CK), optimal interval fertigation (OIF) and optimal daily fertigation (ODF) on cucumber economic yield, irrigation water use efficiency (IWUE) and partial factor productivity of applied nitrogen (PFP_N_) in the early-spring (ES) and autumn-winter (AW) seasons.

Cropping season	Treatment	Economic yield (t ha^−1^)	IWUE (kg mm^−1^)	PFP_N_ (kg kg N^−1^)
ES	CK	132.6b^a^	379. 8b	161. 9c
	OIF	130.2b	372. 8b	304. 5b
	ODF	140.8a	403. 1a	329. 2a
AW	CK	41.2b	279. 8b	88. 6c
	OIF	41.5b	281. 7b	273. 7b
	ODF	44.6a	303. 2a	294. 5a

a The same letter in the same data column denotes no significant difference (P≤0.05) by LSD.

### Vitamin C, soluble sugar and nitrate in cucumber fruit

In general, there was no significant (*P*>0.05) difference in soluble sugar in cucumber fruit between the optimal fertigation treatments (i.e. OIF and ODF) and the CK (Table 6). However, the nitrate in cucumber fruit was significantly (*P*<0.05) decreased by the treatment OIF in the ES and AW seasons, and by the treatment ODF in the AW season, when compared to the CK. In addition, vitamin C in cucumber fruit was significantly (*P*<0.05) increased by the treatment ODF in both ES and AW seasons, when compared to the treatment OIF.

**Table 6:** Effects of conventional interval fertigation (CK), optimal interval fertigation (OIF) and optimal daily fertigation (ODF) on the contents of vitamin C, soluble sugar and nitrate in cucumber fruit in the early-spring (ES) and autumn-winter (AW) seasons.

Cropping season	Treatment	Vitamin C (mg 100 g^−1^)	Soluble sugar (mg g^−1^)	Nitrate (ug g^−1^)
ES	CK	6. 94ab^a^	20. 78a	92. 23a
	OIF	6. 71b	19. 81a	76. 97b
	ODF	7. 31a	21. 12a	84. 94ab
AW	CK	5. 51ab	32. 56a	137. 68a
	OIF	5. 33b	32. 31a	78. 65b
	ODF	5. 76a	31. 02a	63. 89b

a The same letter in the same data column denotes no significant difference (P≤0.05) by LSD.

## Discussion

### Effects of different fertigation treatments on soil water and nutrients

The spatial and temporal distribution of water and nutrients is very heterogeneous in soil. Plant root growth not only can be induced by nutrient supply intensity on the whole, but also can be effected by spatiotemporal variation of water and nutrients [31], [32]. Thus, it is important to maintain a relative stable nutrient supply in plant root-zone. Our results clearly showed that increasing the frequency of fertigation could decrease the amplitudes of water and nutrient contents in soils (Table 2). In general, optimal daily fertigation significantly decreased the amplitudes of water content, EC value and mineral N content in the 0–15 cm soil layer in the AW season, when compared to optimal interval fertigation (ODF *vs* OIF; Table 2). Thus, the water and nutrient limitation for plant root growth is probably not likely in soils under optimal daily fertigation. In addition, since the topsoil (0–15 cm) received more water and nutrient than the subsoil (15–45 cm) under fertigation conditions, optimal daily fertigation significantly promoted the root length of <1.5 mm diameter in the 0–15 cm soil layer when compared to optimal interval fertigation (ODF *vs* OIF; Fig. 4). These results are in accordance with previous researches [33]–[36] and further demonstrate that using the traditional interval fertigation may lead to larger fluctuation of water and nutrients in soil and inhibit plant root growth [37], [13]. Furthermore, since soil relative water contents in the 0–15 cm soil layer generally exceeded 25.5% and were higher than soil field capacity (20.8%) under optimal daily fertigation in the AW season (Fig. 3), there is a possibility for our study to further reduce the irrigation rate.

### Effects of different fertigation treatments on plant root growth

Plant roots are able to make different responses to different water and nutrient contents of soils. Although excessive fertilizer application can increase crop yield, it can inhibit plant root growth due to the high nutrient concentration in root-zone. Furthermore, unused nutrients will accumulate in the soils and finally enhance the potential threat to the environment. In contrast, root growth can be depressed when soil nutrient is in deficit [38], [2], [39], [40]. In general, optimal fertilizer application can promote plant root growth and improve fertilizer use efficiency. In this study, however, optimal fertilizer application had no promoting effect on both cucumber root growth (Fig. 4B) and economic yield (Table 5) in the AW season under same fertilizer application ways (OIF *vs* CK). The explanation is that although the amount of inorganic fertilizer application was sufficient for plant root growth under the treatment OIF during middle fruit harvest period, it was insufficient for plant root growth during the late fruit harvest period, resulting in significant inhibition of root growth and no significant effect on the cucumber economic yield. For instance, contents of mineral nitrogen in soil laye of 0–30 cm of the treatments CK, OIF and ODF during the late fruit harvest period were respectively 93, 35 and 61 mg kg^−1^. The contents of mineral nitrogen of the treatment OIF was obviousely less than 50 mg kg^−1^, which is the lower limit of soil N_min_ content for depth 0–30 cm for normal growth and development of cucumber [41], leading to significantly inhibit root growth in the AW seasnon. The results presented here suggested that fertilizer should be applied according to the actual nutrient requirement of the crops at different growing stages.

### Effects of different fertigation treatments on cucumber fruit yield

Root growth is closely coordinated with shoot growth [42]. Generally, spatial distribution of roots in soils, together with distribution of water and nutrients in root-zone and water and nutrients requirements for root and shoot growth, determine the water and nutrient use efficiency of crops and the economic yield formation [43]. The data on economic yield in this study showed that although the amount of fertilizer applied to soil was visibly lower under optimal interval fertigation than under conventional interval fertigation (Table 1), optimal interval fertigation still met the water and nutrient requirements of cucumber (Fig. 3) and maintained root growth (Table 3 and Fig. 4), resulting in the higher efficiency of roots as absorbing organs and no significant effect on cucumber economic yield (Table 5). Thus, excessive fertilizer applied to soil under conventional interval fertigation did not make much contribution to increasing crop yield. In contrast, it can inhibit plant root growth due to the high nutrient concentration in root-zone.

Although soils under the treatments OIF and ODF received the same amount of fertilizer (Table 1), the treatment ODF significantly increased economic yield when compared to the treatment OIF (Table 5). This can be explained by the fact that ODF maintained sufficient and relatively stable water and nutrients in soil (Table 2) and promoted proliferation of fine roots which increased root surface area (Table 3). In addition, ODF reduced the spatiotemporal variation of water in root-zone when compare to OIF (Fig. 3). It was also supported by two recent studies: Gao et al. [14] found that the technology of solution daily application based on the requirement of crops had a promoting effect on tomato yield in a plotted system; Yoshida [44] reported that this technology could increase both the yield of tomato fruit and the water and fertilizers use efficiency by plant.

Root function is related to the morphological and physiological characteristics of root. However, this study did not consider the root physiological function, hence, further work is required test the root physiological function to elucidate the mechanism of communication of root and shoot growth.

### Effects of different fertigation treatments on nitrate content in cucumber fruit

In general, vegetables can provide 80% of nitrate absorbed into the human body [45]. However, part of these nitrate can be reduced to nitrite by bacteria, which may induce lower oxygen-carrying capacity of blood and methemoglobinemia. In addition, nitrite can produce a reaction with secondary amines, such as amides and amino acids, possibly causing the formation of nitrosamines, which may induce cancer in the digestive system of human body [46]. Therefore, it is important to control and maintain low nitrate content in edible part of vegetables. In this study, both OIF and ODF treatments significantly decreased nitrite content, compared to CK treatment. The relative high nitrate content in fruit under CK can be explained by the previous research that surplus nitrate can be partly absorbed and stored by the plant than its need, in order to maintain normal growth requirement when nitrate supply is in deficit [47]. The results presented here suggested that reducing the fertilizer amounts, according to the actual nutrient requirement of the crops at different growing stages, is beneficial to fruit quality and human healthy without having a significant effect on cucumber fruit yield.

## Conclusions

On the premise of optimal management of irrigation rate for cucumber, optimal interval fertilization based on nutrient target values, including soil mineral nutrient safety margin and crop uptake, and soil nutrients in root-zone had no significant effect on economic yield, but increased partial factor productivity of applied N. Optimal daily fertigation based on the actual requirement of crops maintained the relatively stable water and nutrients in soils, reduced the spatiotemporal variation of water and nutrients in root-zone and promoted cucumber fine root (<1.5 mm diameter) growth. In addition, optimal daily fertigation maintained significantly higher cucumber economic yield, irrigation water use efficiency and partial factor productivity of applied N than optimal interval fertigation.
